# A Machine Learning-Based Investigation of Gender-Specific Prognosis of Lung Cancers

**DOI:** 10.3390/medicina57020099

**Published:** 2021-01-22

**Authors:** Yueying Wang, Shuai Liu, Zhao Wang, Yusi Fan, Jingxuan Huang, Lan Huang, Zhijun Li, Xinwei Li, Mengdi Jin, Qiong Yu, Fengfeng Zhou

**Affiliations:** 1 Department of Epidemiology and Biostatistics, School of Public Health, Jilin University, Changchun 130012, China; wyy18@mails.jlu.edu.cn (Y.W.); zjli19@mails.jlu.edu.cn (Z.L.); xinweil20@mails.jlu.edu.cn (X.L.); jinmd19@mails.jlu.edu.cn (M.J.); 2 College of Computer Science and Technology, and Key Laboratory of Symbolic Computation and Knowledge Engineering of Ministry of Education, Jilin University, Changchun 130012, China; lshuai19@mails.jlu.edu.cn (S.L.); wz19980226@163.com (Z.W.); fan_yusi@163.com (Y.F.); q2857866014@163.com (J.H.); huanglan@jlu.edu.cn (L.H.)

**Keywords:** lung cancer, prognosis, machine learning, gender, survival prediction

## Abstract

*Background and Objective*: Primary lung cancer is a lethal and rapidly-developing cancer type and is one of the most leading causes of cancer deaths. *Materials and Methods*: Statistical methods such as Cox regression are usually used to detect the prognosis factors of a disease. This study investigated survival prediction using machine learning algorithms. The clinical data of 28,458 patients with primary lung cancers were collected from the Surveillance, Epidemiology, and End Results (SEER) database. *Results*: This study indicated that the survival rate of women with primary lung cancer was often higher than that of men (*p* < 0.001). Seven popular machine learning algorithms were utilized to evaluate one-year, three-year, and five-year survival prediction The two classifiers extreme gradient boosting (XGB) and logistic regression (LR) achieved the best prediction accuracies. The importance variable of the trained XGB models suggested that surgical removal (feature “Surgery”) made the largest contribution to the one-year survival prediction models, while the metastatic status (feature “N” stage) of the regional lymph nodes was the most important contributor to three-year and five-year survival prediction. The female patients’ three-year prognosis model achieved a prediction accuracy of 0.8297 on the independent future samples, while the male model only achieved the accuracy 0.7329. *Conclusions*: This data suggested that male patients may have more complicated factors in lung cancer than females, and it is necessary to develop gender-specific diagnosis and prognosis models.

## 1. Introduction

Lung cancer is one of the leading causes of cancer deaths, and it is estimated to have caused 142,670 deaths in 2019 alone [1]. The incidence rate of lung cancer in females is higher than in males [2,3,4]. The worldwide incidence rate of female lung cancers is still increasing [5]. However, the mortality rate of male lung cancer patients is almost twice that of females [6,7].

Inherent genetic factors and living environments induce apparent gender-specific biological mechanisms and prognostic responses [8]. Non-small cell lung cancers (NSCLC) patients demonstrate large gender-specific survival differences [9]. Zang et al. suggested that the gender variation cannot be explained by the differences in the factors of baseline exposure, smoking history, or body size, but may be caused by the higher susceptibility to tobacco carcinogens in females [10]. That is to say, females are more susceptible to tobacco-induced carcinogenesis than males [11]. Gasperino’s study showed that after taking into account the number of cigarettes smoked, females have a three-times higher risk of lung cancer than males [12]. Another study suggested that females have a better survival rate than males after considering confounders like smoking [13]. Many studies observed the gender differences in urothelial carcinoma of the bladder (UCB), where males had a higher incidence of UCB, but females tended to have worse outcomes [14]. Gender was also found to be a risk factor for the prognosis of head and neck cancer (HNC), and females had a better prognosis than males [15]. So, the gender specificity of lung cancers is attracting more researchers to work on this interesting topic.

Cancer prognosis is mainly predicted based on the clinician’s professional experience or the nomogram calculation. The corresponding scores on the upper dots of each variable graph in the nomogram are added up to obtain the total score, and then a straight line is drawn at the bottom of the chart to estimate the probability of death [16,17]. The nomogram becomes very complicated if multiple variables are included [18].A nomogram is a statistical model for the probability calculation of a single event like death or recurrence and has been widely used to predict the probabilities of cancer metastasis and prognosis [19,20,21,22,23]. The non-negligible proportions of the available nomograms shared similar end-points and intrinsic complexity, which may limit their applications [24].Both nomogram and machine learning techniques can be used to estimate the overall survival rate of cancer patients. However, studies have shown that machine learning models outperformed the nomograms in estimating the individual patient’s prognosis [25]. The machine learning model may provide complementary information for this challenge by fully utilizing the inter-variable interactions [26].

This study hypothesizes that the gender disparity should be taken into account when a prognosis model is optimized. Multiple classification algorithms were utilized to build binary classification models of whether a patient may survive one year, three years, or five years after the clinical data were collected. In this case, we used the Cox regression model to analyze the prognosis of lung cancer patients. The classification models were built for females and males separately.

## 2. Materials and Methods

### 2.1. Data Sources

The clinical data used in this study were retrieved from the Surveillance, Epidemiology, and End Results (SEER) database (AYA site recode/WHO 2008 8.3 Carcinoma of trachea, bronchus, and lung) [27,28]. AYA is a site/histology recode used to analyze data on adolescent and young adults. The recode was applied to all cases no matter the age in order that age comparisons can be made with these groupings. For more information, see http://www.seer.cancer.gov/ayarecode/index.html. The SEER database is an authoritative cancer statistical database in the United States, and global cancer researchers may obtain data through application. A signed SEER data usage agreement is required to obtain the fields and variables in the SEER database. Researchers may make scientific investigations into the SEER data and publish research articles based on the analysis of this data. The database is available at https://seer.cancer.gov/. This study included patients diagnosed from 2010 to 2015 and followed up until December 2016 with primary lung cancer. Only the cases with primary tumors and which were positively followed (excluding “necropsy only” and “death certificate only”) were used. The cases with a follow-up time of 0 may indicate in-hospital deaths and were also excluded. All the objective characteristics of the included patients were collected, including marital status, race, gender, age, histological type, tumor-specific death status, survival time, primary site surgery, tumor grade, lymph node examined, lymph node positive, tumor grade, laterality, year of diagnosis, T stage, M stage, and N stage.

This study collected a cohort of 28,458 patients with primary lung cancers from the SEER database, and most of the patients were women (15,000, 52.71%). Table 1 summarized the baseline characteristics of the primary cancer patients. Statistical significances were observed for gender-specific features, including histological type, marital status, race, primary site surgery, grade, T stage, N stage, and M stage.

This dataset consisted mostly of the white population, and the observations of this study may serve as a good reflection of the gender-disparity in white lung cancer patients. Table 1 showed that 83.20% of male and 82.50% of female patients were white. 

It is also interesting to observe that female lung cancer patients tended to be diagnosed at an earlier stage than male patients, since more female patients were diagnosed at the smallest T (Tumor)/N (Node)/M(Metastasis) stages than male patients. 45.30% of the female patients were diagnosed at the T1 stage, compared with 36.60% of the male patients. The female lung cancer patients contained percentages of 74.20% and 95.20% with diagnosis at the N0 and M0 stages, while the male patients contained 68.90% and 94.00%, respectively. Moreover, all the T/N/M stages demonstrated statistically significant differences between females and males.

Diagnosed numbers of lung cancer patients were on the rise between 2010 and 2015. However, the relative percentages of females and males were similar and the differential analysis did not show a statistical significance (*p*-value = 0.682).

### 2.2. Preprocessing of the SEER Database

A preprocessing step was carried out on the lung cancer dataset from the SEER database. This study excluded the unknown data entries from the patients’ clinical measurements. The T/N/M stages were annotated and extracted from the SEER database [29,30]. The log odds of the positive lymph nodes (LODDS) were defined as the logarithm of the ratio between the probability of being a positive lymph node and the probability of being a negative lymph node. The formula is: LODDS is equal to log (P + 0.5)/(T − P + 0.5), where T is the number of total nodes and P is the number of positive nodes [31]. Marital status was grouped as married and unmarried, where unmarried persons consisted of single, separated, divorced, widowed, and other cases. The differentiation grading codes of 1–4 were defined by the International Classification of Diseases-O-2 (ICD-O-2), where grades I/II/III/IV corresponded to the statuses of well-differentiated, moderately differentiated, poorly differentiated, and undifferentiated, respectively. The histological groups of lung cancers were defined using the ICD-O-3, consisting of adenocarcinoma(8050, 8140-1, 8144, 8201, 8250-5, 8260, 8290, 8310, 8323, 8333, 8470, 8480-1, 8490, 8503, 8507, 8550, 8570, 8574, 8576), squamous cell carcinoma (8051-2, 8070-4, 8083-4, 8123), large cell carcinoma (8012-4, 8021, 8034, 8082), small cell carcinoma (8041-5), and other (8000-1, 8003-4, 8010-1, 8020, 8022, 8030-3, 8035, 8046, 8200, 8230, 8240-1, 8243-6, 8249, 8430, 8525, 8560, 8562, 8575) [2].

Before the data were loaded to the machine learning algorithms, the unordered features were encoded by the one-hot encoding strategy, and the ordered features and numerical features were normalized. One-hot encoding converts categorical variables into numeric variables, e.g., encoding the categorical variable {A, B, C} as the binary variable {100, 010, 001} [32]. Patients were excluded if they had died from causes other than lung cancer, in order to ensure that the predicted survival focused on lung cancers. A supervised classification study needs to know the category labels of the samples, so this study chose to investigate whether the lung cancer patients survived one year, three years, and five years, respectively. For the one-year survival prediction problem, patients were excluded from the dataset if their follow-up lengths were shorter than one year and they were still alive, since we could not know whether such patients died or were still alive one year after the diagnosis. Similar exclusion rules were carried out for the three-year and five-year survival prediction problems.

### 2.3. Binary Classification Algorithms

Seven popular classification algorithms were utilized to build the binary classification models for the one-year, three-year, and five-year survival prediction problems.

Naïve Bayes (NBayes) assumed inter-feature independence and calculated the class-specific prediction model using the Bayes’ theorem. Although NBayes makes a strong assumption of inter-feature independence, it has been widely used to build accurate prediction models [33,34].

Three tree-based classifiers were evaluated for the survival prediction problems in this study. The decision tree (DTree) is a fast and popular classifier, and its trained tree structure is easy to be interpreted [35]. Random forest (RF) is an ensemble algorithm based on multiple random trees as the base classifiers, and has demonstrated its efficiency for biomedical prediction problems [36]. XGBoost is a popular fast classifier to handle various biomedical data types, including the spectroscopy spectrum and bioelectrical data [37,38].

The simple but effective classifier k-nearest neighbor (KNN) is a supervised learning algorithm based on the voting results of the training samples most similar to the query sample [39].

Logistic regression (LR) calculates the probability of a specific event such as the survival of a lung cancer patient, and is popular for building biomedical prediction models [40,41].

Support vector machine (SVM) tries to find a hyperplane with the largest distance or margin to the two classes of samples in the multi-dimensional space [42].

### 2.4. Evaluation Metrics of Binary Classification Algorithms

The machine learning models were evaluated by four binary classification performance metrics, i.e., sensitivity (Sn), specificity (Sp), accuracy (Acc), and F1 score (F1). Sn and Sp are defined as the percentages of positive and negative samples that were predicted correctly, respectively. Acc refers to the ratio of the number of correctly predicted samples to the total number of all the samples. F1 is an index used to measure the accuracy of a binary classification model in statistics. It takes into account both the accuracy and recall of the classification model. All the four performance metrics are from 0 to 1, with higher values indicating better classification performance.

### 2.5. Data Analysis Procedure

**Figure 1:**
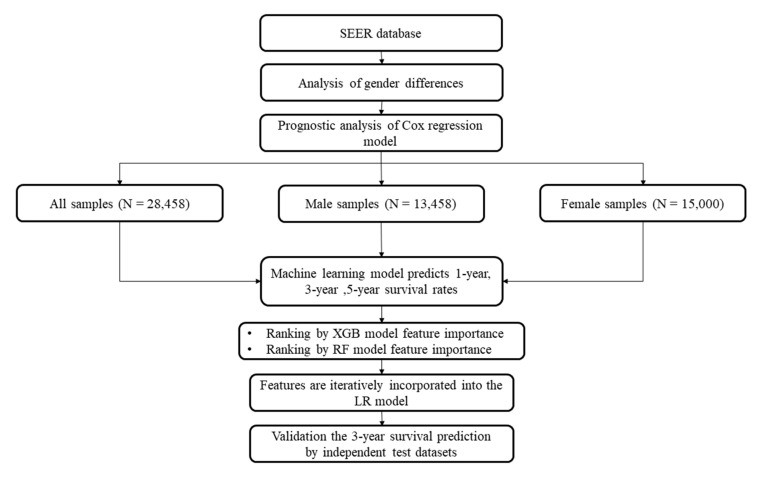
Flow chart of this study. The male and female samples were grouped as the datasets “Male” and “Female”, respectively. All the samples constitute the overall dataset “All”. The sample numbers are provided in parenthesis.

The data analysis process is shown in Figure 1. The baseline data analysis formulated the categorical data as percentages and the continuous data as averaged values and standard deviations. Two statistical tests, Chi-squared test (Chi2test) and *t*-test (Ttest), were used to evaluate the associations between the category or continuous features and gender information. The log-rank test was used on the two genders with the clinical data from the KM chart, which is a letter chart based on the Monoyer standard [43]. The Cox regression was used to perform univariate survival analysis of the individual features. All the prognostic factors derived from the univariate analysis were collected for the multivariate analysis. We also used the nomogram analysis method based on the COX risk ratio model to make predictions. This study defined a survival probability of 1 year, 3 years, and 5 years score less than 0.5 as death, and more than 0.5 as alive.

The classification model was evaluated by the stratified three-fold cross-validation strategy (S3FCV). S3FCV means that the positive and negative samples were randomly split into three equally-sized sub-groups, respectively. One positive subset and one negative subset were used as the test dataset and the other samples were used to train the classification model. This process was iteratively conducted until no sample subset was used as the test dataset. The overall prediction performance metrics were calculated for this iteration [44]. The parameters of all the utilized machine learning models are shown in Supplementary Table S3. All the calculations and experiments were performed using SPSS software version 24.0 and Python version 3.6. The machine learning algorithms were implemented in the Python module scikit-learn version 0.19.1. The multivariate survival analysis was conducted using the Cox regression model.

## 3. Results

### 3.1. Gender Disparities in the Prognosis of Primary Lung Cancers

**Figure 2:**
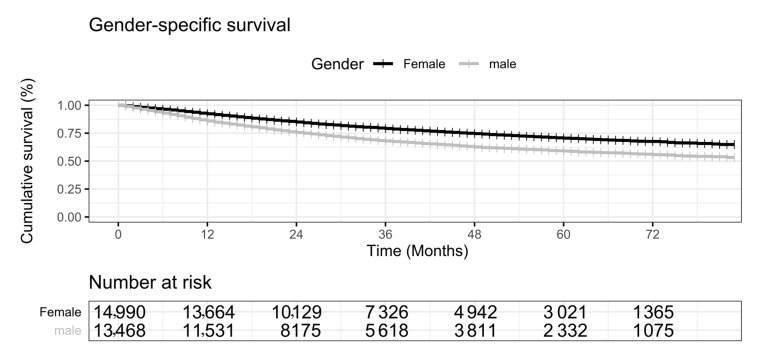
KM (Kaplan-Meier) chart illustration of gender disparity in the survival curves for primary lung cancers. The horizontal axis is the number of months. The vertical axis is the cumulative survival percentage (%). Female is in black, and male is in gray. The numbers of survived patients are given in the table under the plot.

The experimental data suggested that lung cancer patients’ survival probabilities are significantly different between the two genders. We used the log-rank test to measure the difference in survival rates between female and male patients with primary lung cancer (*p* < 0.001). The female patients with primary lung cancer tended to have a better survival rate than male patients (Figure 2). The one-year survival rate of male patients was 85.9%, while the female patients had a survival rate of 92.4%. The female patients had better survival rates 79.1% and 70.5% at 3 years and 5 years, compared with 68.0% and 59.0% for the male lung cancer patients.

The Cox regression was used to evaluate the prognosis of the investigated features, as shown in Table 2. The univariate analysis suggested that gender, age, and race had significant effects on prognosis. For clinicopathological factors, LOODS, histological type, grade, T, N, M, and primary site surgery are all prognostic factors affecting lung cancer (Table 2). Laterality has no effect on the prognosis of lung cancer patients (HR (Hazard Ratio) = 1.023, *p*-value = 0.322). The above-mentioned prognostic-related factors are included in the multivariate analysis. Gender was identified as an independent prognostic factor with an adjusted hazard ratio of 0.698 (95%CI 0.666,0.731). The risk of death without surgery in the primary site was 1.631 times higher than for surgery (*p*-value < 0.001). On the contrary, marriage is a protective factor for primary lung cancer (HR = 0.865, *p*-value < 0.001). T stage, N stage and M stage were independent risk factors for prognosis, among which M = 1 was 2.137 times higher than that of M = 0 (*p*-value < 0.001).

### 3.2. Machine Learning-Based Prediction of Survival Status

**Figure 3:**
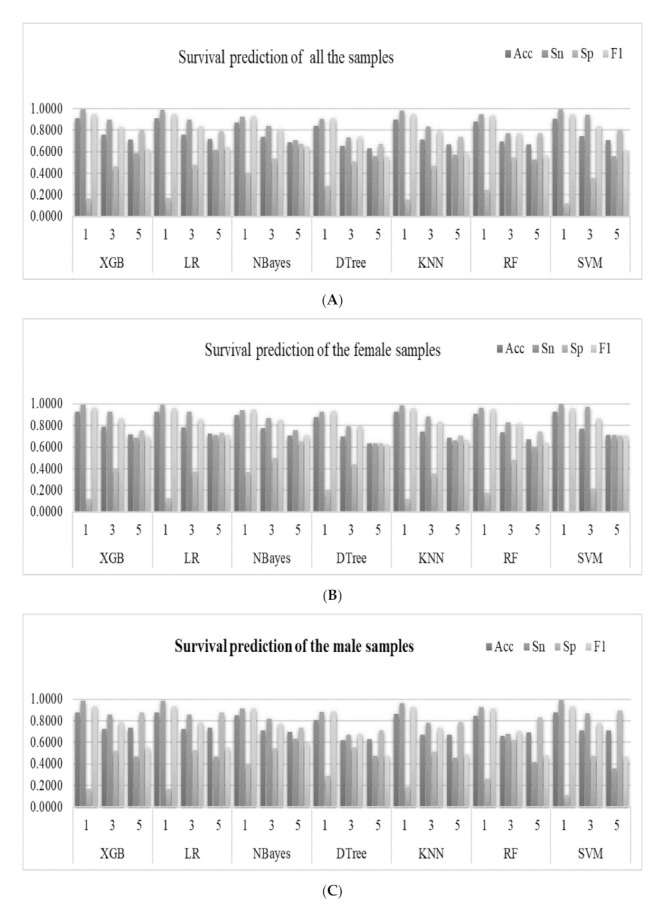

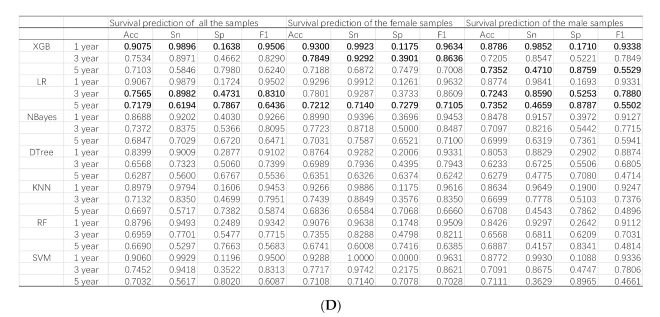
Predictions of the survival status of primary lung cancer patients using machine learning models. The horizontal axis listed the prediction results of the seven binary classifiers for the one-year, three-year and five-year survival prediction problems, which were denoted as 1, 3 and 5, respectively. The four classification performance measurements Acc/Sn/Sp/F1 were calculated for (**A**) all the samples, (**B**) female samples, and (**C**) male samples. (**D**) The detailed data are also provided in this table. The best values for the one-year, three-year, and five-year prediction problems are highlighted in bold, respectively.

Seven popular binary classifiers were utilized to predict whether a lung cancer patient survived for one, three, and five years, as shown in Figure 3. Figure 3A illustrates the machine learning models of the survival prediction problems for all the samples. The classifier XGB achieved the highest Acc for the one-year survival prediction problem (Acc = 0.9075). Another classifier LR achieved the highest Acc values for the three-year (Acc = 0.7565) and five-year (Acc = 0.7179) survival prediction problems.

The machine learning models performed slightly differently on the gender-specific datasets, as shown in Figure 3B,C. The classifier XGB achieved the highest Acc = 0.8786 for the one-year survival prediction problem for the male samples. Similar to the above, the classifier LR achieved the highest Acc for the three- (Acc = 0.7243) and five-year (Acc = 0.7352) survival prediction problems, as shown in Figure 3C. The classifier XGB achieved the highest Acc for the one- (Acc = 0.9300) and three-year (Acc = 0.0.7849) survival prediction problems. Similarly, the classifier LR achieved the highest Acc for the five-year (Acc = 0.7212) survival prediction problems, as shown in Figure 3B.

The nomogram in the survival analysis was based on the COX risk ratio model. Therefore, we used the COX risk ratio model to predict survival at one, three, and five years. In experimental data for all the samples (denoted as “Total”), compared with the COX-based nomogram model, XGB achieved better results in one-year and three-year survival predictions, and LR has a higher accuracy rate in five-years, as shown in Figure 3 and Supplementary Table S1.

### 3.3. Feature Contributions of the XGB Models

The feature contribution was measured by the importance of each feature returned by the trained XGB models for the one-year, three-year, and five-year survival prediction models (Table 3). Except for the feature “Gender” for the dataset of all the samples (denoted as “All”), all the features were ranked in the descendent order of their XGB model importance measurements. The results for the datasets of male and female samples were denoted as “Male” and “Female”.

**Figure 4:**
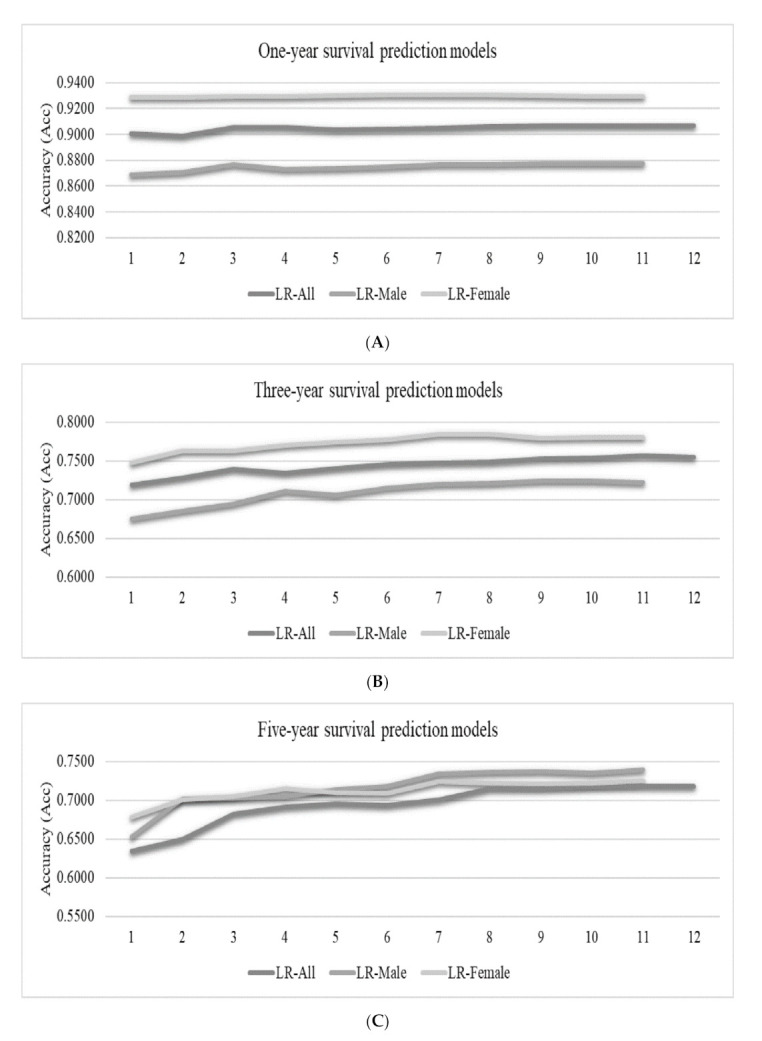
Accuracies (Acc) of the best-performing logistic regression (LR) classifier for the survival prediction problems. The vertical axis listed the feature ranks, which varied for different datasets. Each number k gave the prediction accuracies of the top-k ranked features. The vertical axis gave the prediction accuracies for (**A**) the one-year, (**B**) three-year and (**C**) five-year survival prediction problems.

We further evaluated how importantly each feature contributed to the classification models, as shown in Figure 4. The above sections demonstrated that the two classifiers XGB and LR usually performed best on the one-year, three-year and five-year survival prediction problems. However, the classifier LR did not generate a measurement of feature importance, so the measurement of feature importance from the trained XGB models was used to describe each feature’s contribution to the prediction models.

Figure 4A suggests that the feature “Surgery” is the most important factor for one-year survival, while the spreading of the lung cancers to the regional lymph nodes (the N stage) played an essential role in determining whether a lung cancer patient may survive for three and five years, as shown in Figure 4B,C. The T/N/M stages (features “T”, “N” and “M”) and the grade (feature “Grade”) were consistently ranked after the feature “Surgery” for all the three datasets “All”, “Male” and “Female” for the one-year survival prediction problem. The top-five ranked features were the same for the three-year and five-year survival prediction problems, as shown in Figure 4B,C. The XGB models were constructed to predict the survival status of patients with primary lung cancers and visualized for easy inspection by the reader (Supplementary Figure S1).

**Figure 5:**
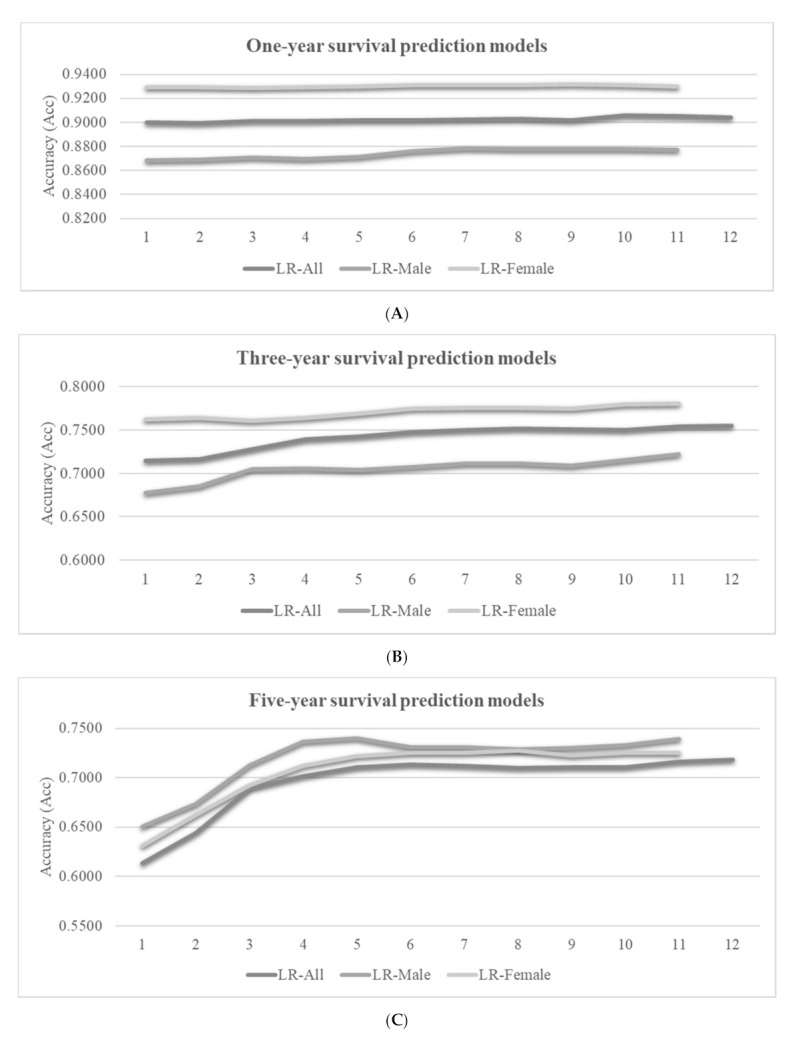
Accuracies (Acc) of the best-performing classifier LR for the survival prediction problems. The vertical axis lists the element level of the random forest (RF) model arrangement, which varied for different datasets. Each number k gave the prediction accuracies of the top-k ranked features. The vertical axis gave the prediction accuracies of (**A**) the one-year, (**B**) three-year and (**C**) five-year survival prediction problems.

The LR classifier was used to calculate the accuracies of the one-year, three-year, and five-year survival prediction problems, as shown in Figure 5. The features were incrementally added to the feature subsets by their ranks calculated in the previous section. Since the datasets did not have consistent ranks for the features, Figure 5 only gives the feature ranks in the horizontal axis.

We may observe the overall trend that more features may achieve better prediction accuracies, as shown in Figure 5. However, the inclusions of some features may decrease the model performance in some cases. For example, the best LR-Female model achieved Acc = 0.9309 using only six features, and the model accuracy was decreased to Acc = 0.9296 by using five more features. The survival prediction model usually achieved a very good prediction accuracy using about six features, and the inclusions of more features only achieve minor accuracy improvements.

### 3.4. Feature Contributions to the RF Models

The machine learning algorithm RF also calculated the importance measurement of each feature in the one-year, three-year, and five-year survival prediction models to measure feature contribution, as shown in Table 4. At the same time, we use the RF model to describe each feature’s contribution to the prediction model for feature importance. The results showed that LOODS and Age were always the most important factors for survival status in one, three, and five years. This observation was slightly different to those features with large contributions in the XGB models. We added the features incrementally to the feature subsets. We also found that the best LR-Female model can reach Acc = 0.9315 using only nine features and adding two features reduces the accuracy of the model to Acc = 0.9296 (Figure 5). Studies have shown that there are multiple optimal solutions for the same problem [45]. In the future, when we are looking for cancer markers, we can consider different combinations of features.

### 3.5. Independent Validation of the Models Using Future Samples

This study validated only the three-year survival prediction models due to the limited numbers of diagnosis years in the SEER database. The samples from the diagnosis years 2010–2011 were used as the training samples. Moreover, the samples from 2012–2013 were the independent validation samples. The survival statuses of the samples diagnosed in 2013 were determined by the follow-up data in the years 2014–2016, where the 2016 data were also retrieved from the SEER database. It is interesting to observe that the LR model was trained on the 2010–2011 samples and achieved Acc = 0.7841 on the “All” dataset from the diagnosis years 2012–2013. The female samples demonstrated an even better consistency in the three-year survival prediction by the independent validation with Acc = 0.8297. The male samples received a slightly worse model with Acc = 0.7329. We found that most pairs of investigated baseline characteristics did not have statistically significant and strong correlations (correlation coefficient > 0.300 and *p* < 0.05), except for a few cases. The variables N and LOODS were observed to have a correlation coefficient of 0.630 and *p* < 0.001. The variable Surgery was correlated with N (correlation coefficient = 0.323, *p* < 0.001) and M (correlation coefficient = 0.348, *p* < 0.001). The variable Stage was correlated with T (correlation coefficient = 0.653, *p* < 0.001), LOODS (correlation coefficient = 0.371, *p* < 0.001), N (correlation coefficient = 0.579, *p* < 0.001), and M (correlation coefficient = 0.415, *p* < 0.001). The other pairs of these baseline characteristics were either not or were weakly correlated (Supplementary Table S2). The machine learning models developed in the above sections suggested that some features’ removal may improve the prognosis prediction models.

## 4. Discussion

Gender disparity was observed in lung cancer incidence, mortality, prognosis and treatment responses, etc. The prognosis analysis in this study suggested that female lung cancer patients had a better prognosis than male patients. Radkiewicz et al. found that the prognosis of male patients with non-small cell lung cancers (NSCLC) was poor, even after careful adjustments for various prognostic factors [46]. This study confirmed this observation and observed the gender-specific developmental T/N/M stages on diagnosis. Biological differences may justify female’s better treatment responses and improved survival rates [47]. Kinoshita et al. also pointed out that gender differences in the histological types and developmental stages on diagnosis may partially explain the better prognosis of female lung cancer patients [48]. Studies of both surgery and systemic therapeutic treatments suggested that, besides the never smokers, female lung cancer patients overall experienced a better prognosis than males [49].

Both inherent genetic factors and gene-environment interactions play important roles in regulating the prognosis of lung cancers [7,50,51]. Various factors may induce the poor prognosis of male lung cancer patients. Firstly, female lung cancer patients benefited more from the anti-programmed cell death-1programmed death ligand-1 (anti-PD-1/PD-L1) chemotherapy than male patients [52]. Immune checkpoint inhibitors were more effective in male patients than female patients, while immune checkpoint inhibitors combined with chemotherapy were often more effective in female patients [53]. Secondly, male lung cancer patients tended to have more aggressive tumor developmental behaviors, e.g., faster growth and higher metastatic potentials [46].

Machine learning algorithms are becoming a popular technique to predict survival status. Compared with nomograms, machine learning models are not easy to be interpreted, but clinical doctors do not have to manually calculate the risk scores as with nomograms. A machine learning model may deliver prediction results rapidly and conveniently to the users once the input variables are loaded. The survival prediction results in this study showed better prediction performances for female patients with primary lung cancers than male patients. The two classifiers XGB and LR performed similarly well on the binary classifications of the one-year, three-year, and five-year survival prediction problems.

The feature contributions to the prediction models were evaluated by the importance variables of the trained XGB models. One-year survival status heavily relied on the variable “Surgery” for both genders and their mixture, while the most important contributions came from the variable “N” stage for the three-year and five-year survival prediction problems. So, a surgical removal is important for the one-year survival of primary lung cancer patients. Then, a long-term goal for primary lung cancer patient is to pay special attention to monitoring the regional lymph nodes for possible metastatic tumor development.

This study has the following limitations, which may be overcome by more comprehensive data sources. The SEER database provides a limited set of clinical variables for the patient and it is anticipated that more clinical data, e.g., on smoking and drinking, will improve the model performances. The imaging and molecular data of the patient’s mental health, genetic information, and living environment will definitely increase the models’ prediction performance. This study selected seven commonly-used machine learning algorithms to investigate the gender-specific prognosis of lung cancers. This gender disparity may be further evaluated by more machine learning algorithms. Novel machine learning algorithms may also be developed in future studies to deliver gender-independent prognosis models with similar prediction performances to the gender-specific models. For example, a combination of nomogram and machine learning prediction models can be considered for analysis. Recently, multiple instance learning has demonstrated its superiority in various applications including tumor imaging analysis [54,55,56,57]. The deployment of the multiple instances learning method may significantly improve prognosis prediction for cancer patients. Spherical separation surface-based approaches were observed to perform better than classifiers based on linear separation surfaces on the binary classification problems of two similar class labels, and may be utilized in future investigations [58,59]. Gender-specific prognosis may be investigated using biomedical imaging data and other data types in future studies.

## 5. Conclusions

In conclusion, this study provided an exploratory investigation of gender disparity in the prognosis of primary lung cancers using regular statistical methods and machine learning prediction models. Both techniques consistently supported the prognosis observations derived from each other. Due to the limited availability of the primary lung cancer dataset with long-term follow-ups, the observations were not validated by an independent dataset.

## Figures and Tables

**Table 1:** Baseline characteristics of the study population. The variable t refers to the test statistics of the *T*-test, and the variable χ^2^ refers to the chi-square value of the chi-square test.

	**Female**	**Male**	**t**	***p*-value**
Age	66.19 ± 10.52	66.45 ± 9.70	2.16	0.03
LOODS	−2.24 ± 1.27	−2.18 ± 1.32	3.77	<0.001
	**Female**	**Male**	**χ^2^**	***p*-value**
Race			10.22	0.006
White	12,362 (82.50%)	11,204 (83.20%)		
Black	1447 (9.70%)	1157 (8.60%)		
Other	1181 (7.90%)	1107 (8.20%)		
Histological Type			836.71	<0.001
Adenocarcinoma	10,256 (68.40%)	7330 (54.40%)		
Squamous cell carcinoma	2646 (17.70%)	4319 (32.10%)		
Large cell carcinoma	239 (1.60%)	265 (2.00%)		
Small cell carcinoma	203 (1.40%)	165 (1.20%)		
Other	1646 (11.00%)	1389 (10.30%)		
Grade			432.92	<0.001
Grade I	3296 (22.00%)	1865 (13.80%)		
Grade II	6522 (43.50%)	5701 (42.30%)		
Grade III	4843 (32.30%)	5580 (41.40%)		
Grade IV	329 (2.20%)	322 (2.40%)		
surgery			50.41	<0.001
YES	14,241 (95.00%)	12,521 (93.00%)		
NO	749 (5.00%)	947 (7.00%)		
Marital status			1017.37	<0.001
Single	7408 (49.40%)	4151 (30.80%)		
Married	7582 (50.60%)	9317 (69.20%)		
Laterality			1.84	0.175
Right	8826 (58.90%)	77,823 (58.10%)		
Left	6164 (41.10%)	5645 (41.90%)		
T			249.96	<0.001
T1	6796 (45.30%)	4931 (36.60%)		
T2	5526 (36.90%)	5462 (40.60%)		
T3	1877 (12.50%)	2244 (16.70%)		
T4	791 (5.30%)	831 (6.20%)		
N			108.54	<0.001
N0	11,116 (74.20%)	9278 (68.90%)		
N1	1740 (11.60%)	1987 (14.80%)		
N2	1934 (12.90%)	1944 (14.40%)		
N3	200 (1.30%)	259 (1.90%)		
M			20.22	<0.001
M0	14,266 (95.20%)	12,655 (94.00%)		
M1	724 (4.80%)	813 (6.00%)		
Year of diagnosis			3.47	0.682
2010	2367 (15.80%)	2176 (16.20%)		
2011	2381 (15.90%)	2164 (16.10%)		
2012	2348 (15.70%)	2169 (16.10%)		
2013	2541 (17.00%)	2216 (16.50%)		
2014	2602 (17.40%)	2332 (17.30%)		
2015	2751 (18.40%)	2411 (17.90%)		

**Table 2:** prognosis of primary lung cancer of different genders.

	**Univariate**		**Multivariate**	
	**HR(95%CI)**	***p*-value**	**HR(95%CI)**	***p*-value**
Age	1.017(1.014,1.019)	<0.001	1.024(1.021,1.026)	<0.001
LOODS	1.505(1.483,1.526)	<0.001	1.179(1.154,1.205)	<0.001
Race		<0.001		0.001
White	1		1	
Black	0.984(0.912,1.063)	0.685	0.998(0.923,1.079)	0.961
Other	0.828(0.759,0.904)	<0.001	0.841(0.77,0.918)	<0.001
Sex		<0.001		<0.001
Male	1		1	
Female	0.628(0.601,0.657)		0.698(0.666,0.731)	
Histological Type		<0.001		<0.001
Adenocarcinoma	1		1	
Squamous cell carcinoma	1.501(1.427,1.578)	<0.001	1.171(1.111,1.235)	<0.001
Large cell carcinoma	2.049(1.792,2.342)	<0.001	1.603(1.389,1.851)	<0.001
Small cell carcinoma	3.381(2.95,3.876)	<0.001	1.481(1.272,1.724)	<0.001
Other	1.086(1.007,1.172)	0.032	1.073(0.994,1.16)	0.072
Grade		<0.001		<0.001
Grade I	1		1	
Grade II	2.455(2.245,2.685)	<0.001	1.747(1.593,1.915)	<0.001
Grade III	4.273(3.914,4.665)	<0.001	2.305(2.102,2.526)	<0.001
Grade IV	5.424(4.713,6.242)	<0.001	2.306(1.97,2.701)	<0.001
surgery		<0.001		<0.001
YES	1		1	
NO	5.354(5.036,5.692)		1.631(1.502,1.77)	
Marital status		0.008		<0.001
Single	1		1	
Married	0.941(0.9,0.984)		0.865(0.826,0.907)	
Laterality		0.322		
Right	1			
Left	1.023(0.978,1.069)			
T		<0.001		<0.001
T1	1		1	
T2	2.068(1.954,2.188)	<0.001	1.529(1.443,1.62)	<0.001
T3	3.5(3.283,3.732)	<0.001	2.23(2.086,2.385)	<0.001
T4	4.633(4.272,5.023)	<0.001	1.992(1.825,2.174)	<0.001
N		<0.001		<0.001
N0	1		1	
N1	2.453(2.312,2.602)	<0.001	1.554(1.452,1.664)	<0.001
N2	3.75(3.557,3.954)	<0.001	1.731(1.606,1.865)	<0.001
N3	8.143(7.307,9.075)	<0.001	1.661(1.443,1.912)	<0.001
M		<0.001		<0.001
M0	1		1	
M1	4.581(4.294,4.887)		2.137(1.984,2.302)	

**Table 3:** Contributions of the investigated features to the extreme gradient boosting (XGB) models.

**One-year**					
**All**		**Male**		**Female**	
**Characteristic**	**Relative Importance**	**Characteristic**	**Relative Importance**	**Characteristic**	**Relative Importance**
Surgery	0.3279	Surgery	0.4734	Surgery	0.2771
T	0.1334	T	0.1284	N	0.1652
M	0.1261	M	0.0972	M	0.1560
N	0.1208	N	0.0801	T	0.1123
Grade	0.0917	Grade	0.0662	Grade	0.0926
Histologic Type	0.0450	Histologic Type	0.0406	LOODS	0.0495
Gender	0.0446	LOODS	0.0375	Histologic Type	0.0471
LOODS	0.0396	Age	0.0289	Age	0.0385
Age	0.0303	Race	0.0217	Marital	0.0294
Marital	0.0174	Marital	0.0155	Race	0.0208
Race	0.0122	Laterality	0.0107	Laterality	0.0115
Laterality	0.0112				
**Three-year**					
**All**		**Male**		**Female**	
**Characteristic**	**Relative Importance**	**Characteristic**	**Relative Importance**	**Characteristic**	**Relative Importance**
N	0.4364	N	0.3591	N	0.4251
Surgery	0.1538	Surgery	0.2031	Surgery	0.1588
T	0.1059	T	0.1508	Grade	0.1152
Grade	0.0883	M	0.0902	T	0.0935
M	0.0695	Grade	0.0633	M	0.0815
Gender	0.0491	LOODS	0.0373	LOODS	0.0308
LOODS	0.0240	Age	0.0290	Histologic Type	0.0278
Histologic Type	0.0235	Histologic Type	0.0256	Age	0.0259
Age	0.0209	Marital	0.0235	Race	0.0169
Race	0.0120	Race	0.0103	Marital	0.0166
Marital	0.0107	Laterality	0.0077	Laterality	0.0080
Laterality	0.0058				
**Five-year**					
**All**		**Male**		**Female**	
**Characteristic**	**Relative Importance**	**Characteristic**	**Relative Importance**	**Characteristic**	**Relative Importance**
N	0.4279	N	0.4389	N	0.4728
Surgery	0.1357	T	0.1282	Grade	0.1299
T	0.1321	Surgery	0.1275	Surgery	0.1144
Grade	0.0858	M	0.0959	T	0.0949
M	0.0523	Grade	0.0611	M	0.0570
Gender	0.0471	LOODS	0.0391	LOODS	0.0316
LOODS	0.0325	Age	0.0341	Age	0.0288
Age	0.0283	Marital	0.0267	Histologic Type	0.0206
Histologic Type	0.0248	Histologic Type	0.0231	Marital	0.0205
Marital	0.0185	Race	0.0150	Laterality	0.0159
Race	0.0079	Laterality	0.0105	Race	0.0135
Laterality	0.0070				

**Table 4:** Contributions of the investigated features to the RF models.

**One-Year**					
**All**		**Male**		**Female**	
**Characteristic**	**Relative Importance**	**Characteristic**	**Relative Importance**	**Characteristic**	**Relative Importance**
LOODS	0.2869	LOODS	0.3040	LOODS	0.2898
Age	0.2820	Age	0.2880	Age	0.2813
T	0.0751	T	0.0814	Grade	0.0802
Grade	0.0664	Grade	0.0738	N	0.0789
N	0.0650	Histologic Type	0.0568	T	0.0768
Histologic Type	0.0537	N	0.0554	Histologic Type	0.0542
Laterality	0.0343	Laterality	0.0357	Laterality	0.0343
Race	0.0326	Race	0.0336	Race	0.0314
Marital	0.0308	Marital	0.0319	Marital	0.0291
Surgery	0.0290	M	0.0210	Surgery	0.0261
Gender	0.0285	Surgery	0.0184	M	0.0179
M	0.0155				
**Three-year**					
**All**		**Male**		**Female**	
**Characteristic**	**Relative Importance**	**Characteristic**	**Relative Importance**	**Characteristic**	**Relative Importance**
LOODS	0.2867	LOODS	0.2993	LOODS	0.2920
Age	0.2594	Age	0.2810	Age	0.2857
T	0.0667	T	0.0732	T	0.0606
Histologic Type	0.0610	Histologic Type	0.0617	Histologic Type	0.0603
N	0.0517	Grade	0.0560	Grade	0.0576
Surgery	0.0515	N	0.0472	N	0.0520
Grade	0.0503	M	0.0409	M	0.0468
Laterality	0.0393	Laterality	0.0384	Laterality	0.0415
Marital	0.0376	Surgery	0.0356	Marital	0.0390
M	0.0341	Race	0.0334	Race	0.0325
Race	0.0318	Marital	0.0332	Surgery	0.0321
Gender	0.0297				
**Five-year**					
**All**		**Male**		**Female**	
**Characteristic**	**Relative Importance**	**Characteristic**	**Relative Importance**	**Characteristic**	**Relative Importance**
LOODS	0.2869	LOODS	0.3040	LOODS	0.2898
Age	0.2820	Age	0.2880	Age	0.2813
T	0.0751	T	0.0814	Grade	0.0802
Grade	0.0664	Grade	0.0738	N	0.0789
N	0.0650	Histologic Type	0.0568	T	0.0768
Histologic Type	0.0537	N	0.0554	Histologic Type	0.0542
Laterality	0.0343	Laterality	0.0357	Laterality	0.0343
Race	0.0326	Race	0.0336	Race	0.0314
Marital	0.0308	Marital	0.0319	Marital	0.0291
Surgery	0.0290	M	0.0210	Surgery	0.0261
Gender	0.0285	Surgery	0.0184	M	0.0179
M	0.0155				

## Data Availability

The data is from SEER database. A SEER Research Data Agreement was obtained to enable access to the SEER data. The database is available at https://seer.cancer.gov/.

## Supplementary Materials

The following are available online at https://www.mdpi.com/1010-660X/57/2/99/s1, Figure S1: Visualization of using XGB model to predict the survival status of patients with primary lung cancer, Table S1: The nomogram predicts the prediction results of 1 year, 3 years, and 5 years of survival, Table S2: Correlation coefficient analysis on the pairs of baseline characteristics, Table S3: Parameters of the 7 machine learning methods.
