# Supplementary material for: A Machine Learning-Based Investigation of Gender-Specific Prognosis of Lung Cancers
Source: Medicina (Kaunas). 2021 Jan 22;57(2):99. doi: 10.3390/medicina57020099 (PMC7911834; doi:10.3390/medicina57020099)
Supplement: Supplementary file 1 [file medicina-57-00099-s001.pdf]

A Machine Learning-based Investigation of Gender-Specific Prognosis of Lung Cancers

Yueying Wang [1], Shuai Liu [2], Zhao Wang [2], Yusi Fan [2], Jingxuan Huang [2], Lan Huang [2], Zhijun Li[1], Xinwei Li [1], Mengdi Jin [1], Qiong Yu [1,*], Fengfeng Zhou [2,*].

[1] Department of Epidemiology and Biostatistics, School of Public Health, Jilin University, Changchun, Jilin Province, China. wyy18@mails.jlu.edu.cn; zjli19@mails.jlu.edu.cn; xinweil20@mails.jlu.edu.cn; jinmd19@mails.jlu.edu.cn; yuqiong@jlu.edu.cn

[2] College of Computer Science and Technology, and Key Laboratory of Symbolic Computation and Knowledge Engineering of Ministry of Education, Jilin University, Changchun, Jilin 130012, China. 2314565235@qq.com ; wz19980226@163.com; fan_yusi@163.com; q2857866014@163.com; huanglan@jlu.edu.cn; ffzhou@jlu.edu.cn

* Correspondence: fengfengzhou@gmail.com (F.Z.); yuqiong@jlu.edu.cn (Q.Y.); Tel.: +86-431-8516-6024 (F.Z.); Fax: +86-431-8516-6024 (F.Z.); Tel.: +86 431 85619451 (Q.Y.)

Supplementary Table S1

The nomogram predicts the prediction results of 1 year, 3 years, and 5 years of survival

		acc	sn	sp	F1
Total	1	0.9061	0.9902	0.1444	0.9500
	3	0.7517	0.9068	0.4416	0.8296
	5	0.7168	0.6149	0.7879	0.6410
Male	1	0.8779	0.9846	0.1693	0.9334
	3	0.7208	0.8595	0.5158	0.7860
	5	0.7339	0.4505	0.8849	0.5405
Female	1	0.9302	0.9928	0.1146	0.9636
	3	0.7795	0.9382	0.3453	0.8618
	5	0.7169	0.7051	0.7279	0.7047

Supplementary Table S2

Correlation coefficient analysis on the pairs of baseline characteristics. The notations *.: p<0.05; **.: p<0.001. The correlation between the two continuous variables Age and LOODS was calculated using the Pearson correlation coefficient. All the other pairs of baseline characteristics were evaluated by the Spearman correlation coefficient.

	Gender	Age	LOODS	Race	Histologic Type	Grade	Surgery	Marital status	Laterality	T	N	M	Stage
Gender	1.000	-0.005	-0.021**	0.008	-0.119**	-0.116**	-0.043**	-0.189**	-0.008	-0.091**	-0.056**	-0.027**	-0.064**
Age	-0.005	1.000	-0.045**	-0.067**	-0.005	-0.011	-0.006	-0.013*	0.003	-0.003	-0.072**	-0.057**	-0.034**
LOODS	-0.021**	-0.045**	1.000	0.030**	0.014*	0.126**	0.282**	-0.002	0.009	0.125**	0.630**	0.183**	0.371**
Race	0.008	-0.067**	0.030**	1.000	-0.075**	-0.017**	0.002	-0.041**	-0.007	0.016**	0.022**	0.004	0.021**
Histologic Type	-0.119**	-0.005	0.014*	-0.075**	1.000	0.206**	0.072**	-0.008	0.031**	0.084**	0.028**	0.019**	0.028**
Grade	-0.116**	-0.011	0.126**	-0.017**	0.206**	1.000	0.147**	-0.028**	0.018**	0.230**	0.221**	0.119**	0.246**
Surgery	-0.043**	-0.006	0.282**	0.002	0.072**	0.147**	1.000	-0.016**	-0.019**	0.203**	0.323**	0.348**	0.279**
Marital status	-0.189**	-0.013*	-0.002	-0.041**	-0.008	-.028**	-0.016**	1.000	0.014*	0.003	0.019**	0.012*	0.008
Laterality	-0.008	0.003	0.009	-0.007	0.031**	0.018**	-0.019**	0.014*	1.000	0.007	0.033**	0.004	0.010
T	-0.091	-0.003	0.125**	0.016**	0.084**	0.230**	0.203**	0.003	0.007	1.000	0.266**	0.200**	0.653**
N	-0.056**	-0.072**	0.630**	0.022**	0.028**	0.221**	0.323**	0.019**	0.033**	0.266**	1.000	0.223**	0.579**
M	-0.027**	-0.057**	0.183**	0.004	0.019**	0.119**	0.348**	0.012*	0.004	0.200**	0.223**	1.000	0.415**
Stage	-0.064**	-0.034**	0.371**	0.021**	0.028**	0.246**	0.279**	0.008	0.010	0.653**	0.579**	0.415**	1.000

Supplementary Figure S1

Visualization of using XGB model to predict the survival status of patients with primary lung cancer. (A), (D) and (G) are visualizations of one-year, three-year and five-year survival prediction problems, respectively. (B), (E) and (H) are visualizations of one-year, three-year and five-year male survival prediction problems, respectively. (C), (F) and (I) are visualizations of female survival prediction problems in one, three and five years respectively. The tree structures were plotted using the function to_graphviz() in the package xgboost version 0.81 in the programming language Python version 3.6.

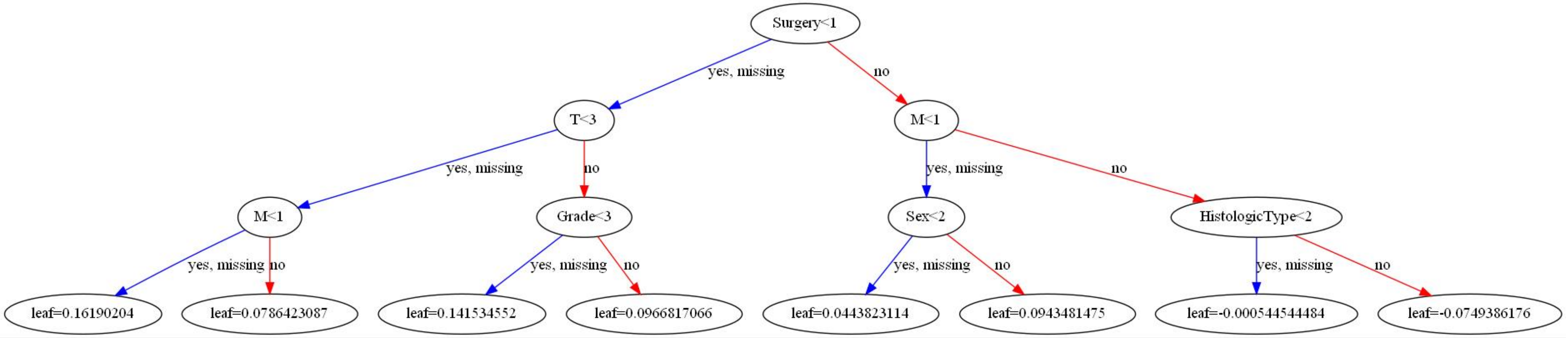

(A)

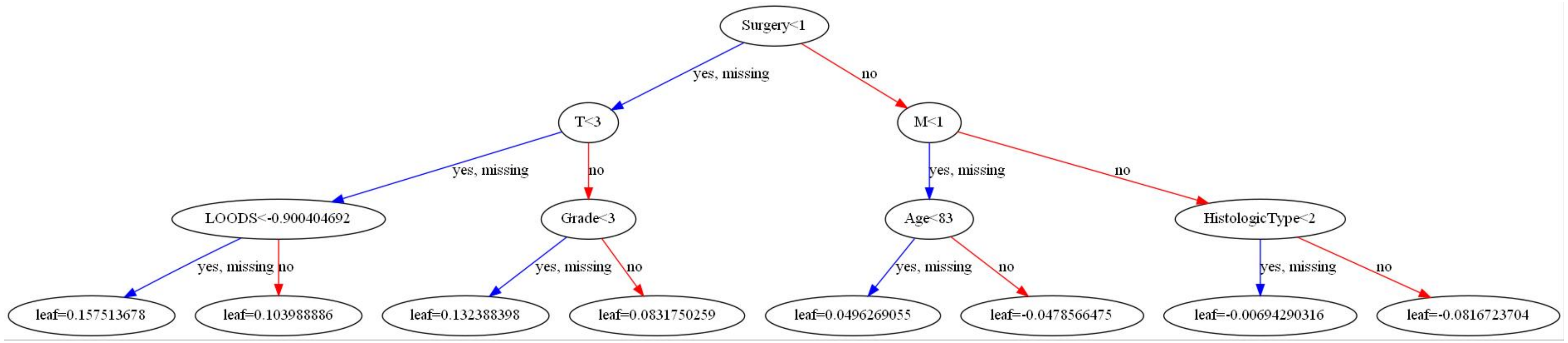
Surgery<1
yes, missing
no
T<3
M<1
yes, missing
no
yes, missing
no
LOODS<-0.900404692
Grade<3
Age<83
HistologicType<2
yes, missing
no
yes, missing
no
yes, missing
no
yes, missing
no
leaf=0.157513678
leaf=0.103988886
leaf=0.132388398
leaf=0.0831750259
leaf=0.0496269055
leaf=-0.0478566475
leaf=-0.00694290316
leaf=-0.0816723704

(B)

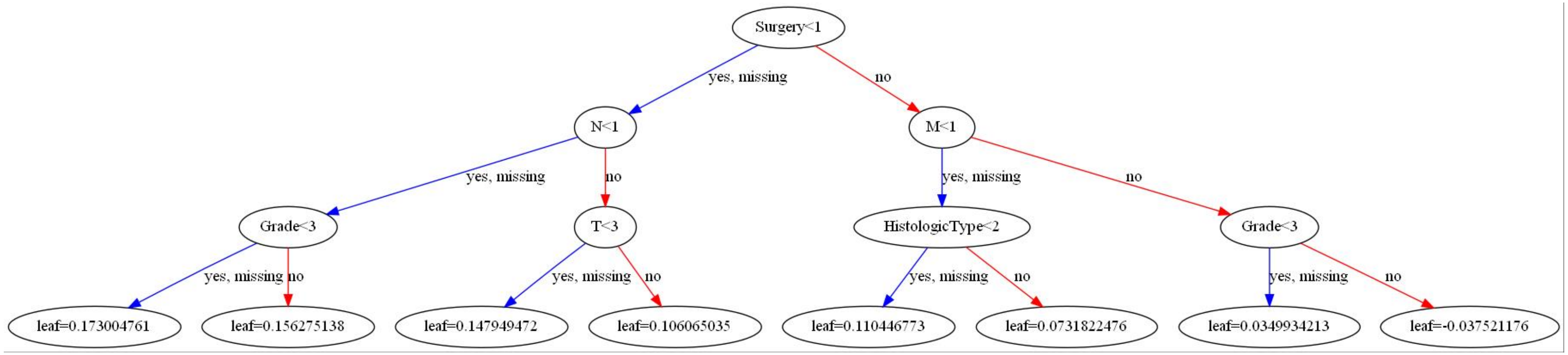
Surgery<1
yes, missing
no
N<1
M<1
yes, missing
no
yes, missing
no
Grade<3
T<3
HistologicType<2
Grade<3
yes, missing
no
yes, missing
no
yes, missing
no
yes, missing
no
leaf=0.173004761
leaf=0.156275138
leaf=0.147949472
leaf=0.106065035
leaf=0.110446773
leaf=0.0731822476
leaf=0.0349934213
leaf=-0.037521176

(C)

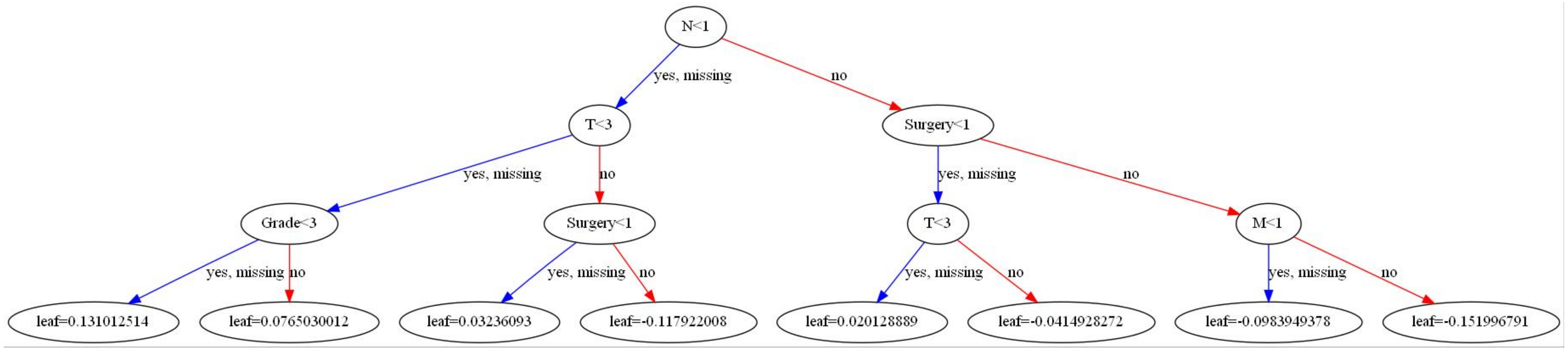
N<1
yes, missing
no
T<3
Surgery<1
Grade<3
Surgery<1
T<3
M<1
leaf=0.131012514
leaf=0.0765030012
leaf=0.03236093
leaf=-0.117922008
leaf=0.020128889
leaf=-0.0414928272
leaf=-0.0983949378
leaf=-0.151996791

(D)

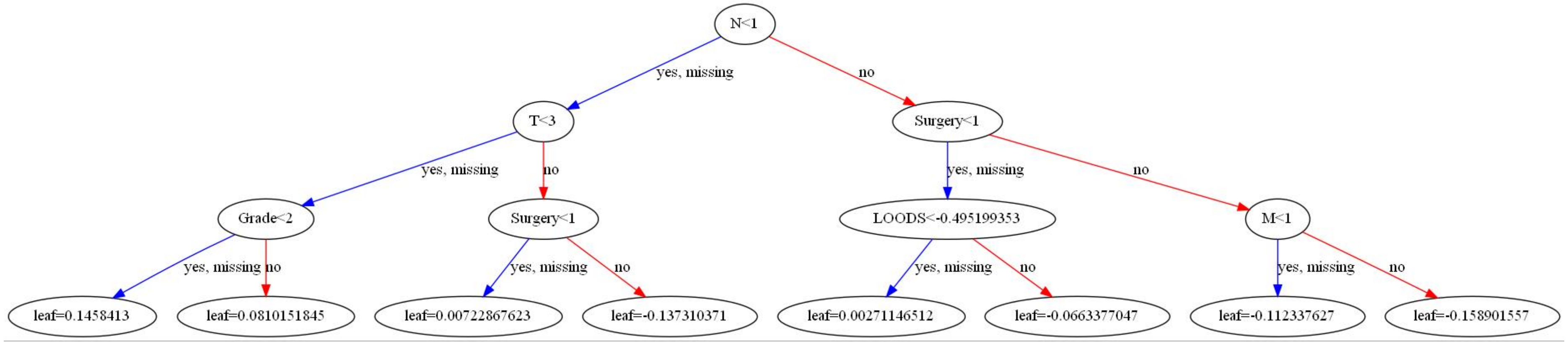
N<1
yes, missing
no
T<3
Surgery<1
Grade<2
Surgery<1
LOODS<-0.495199353
M<1
leaf=0.1458413
leaf=0.0810151845
leaf=0.00722867623
leaf=-0.137310371
leaf=0.00271146512
leaf=-0.0663377047
leaf=-0.112337627
leaf=-0.158901557

(E)

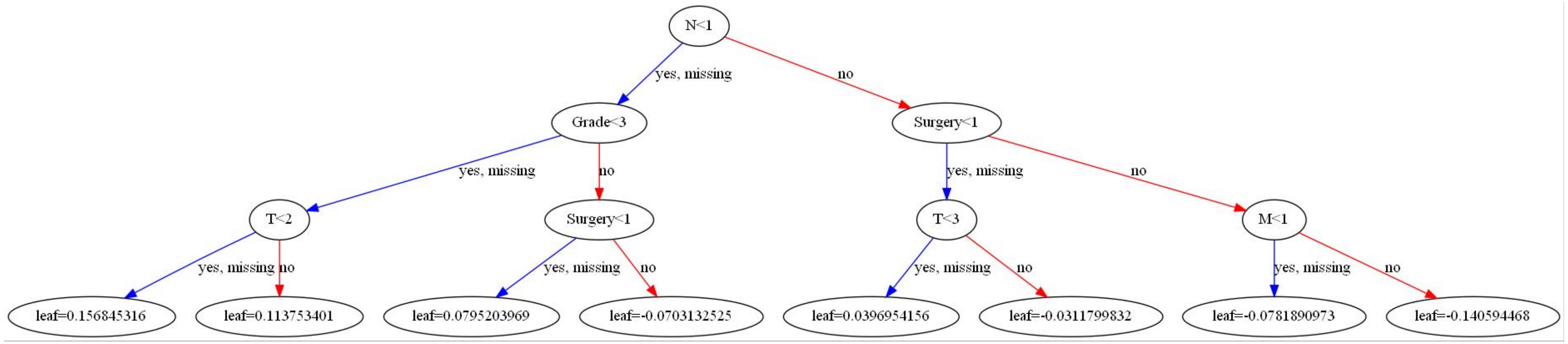
N<1
yes, missing
no
Grade<3
Surgery<1
yes, missing
no
yes, missing
no
T<2
Surgery<1
T<3
M<1
yes, missing
no
yes, missing
no
yes, missing
no
yes, missing
no
leaf=0.156845316
leaf=0.113753401
leaf=0.0795203969
leaf=-0.0703132525
leaf=0.0396954156
leaf=-0.0311799832
leaf=-0.0781890973
leaf=-0.140594468

(F)

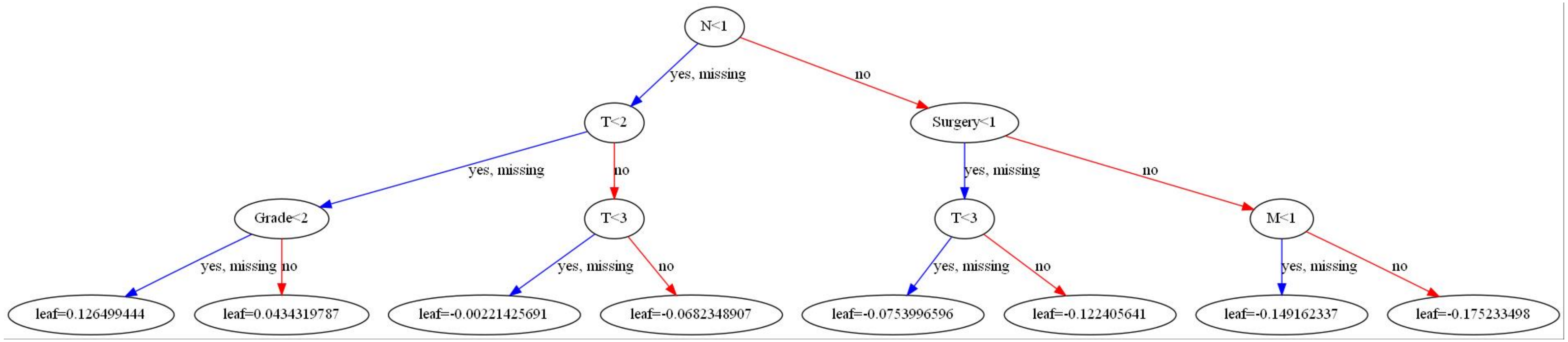
N<1
yes, missing
no
T<2
Surgery<1
yes, missing
no
yes, missing
no
Grade<2
T<3
T<3
M<1
yes, missing
no
yes, missing
no
yes, missing
no
yes, missing
no
leaf=0.126499444
leaf=0.0434319787
leaf=-0.00221425691
leaf=-0.0682348907
leaf=-0.0753996596
leaf=-0.122405641
leaf=-0.149162337
leaf=-0.175233498

(G)

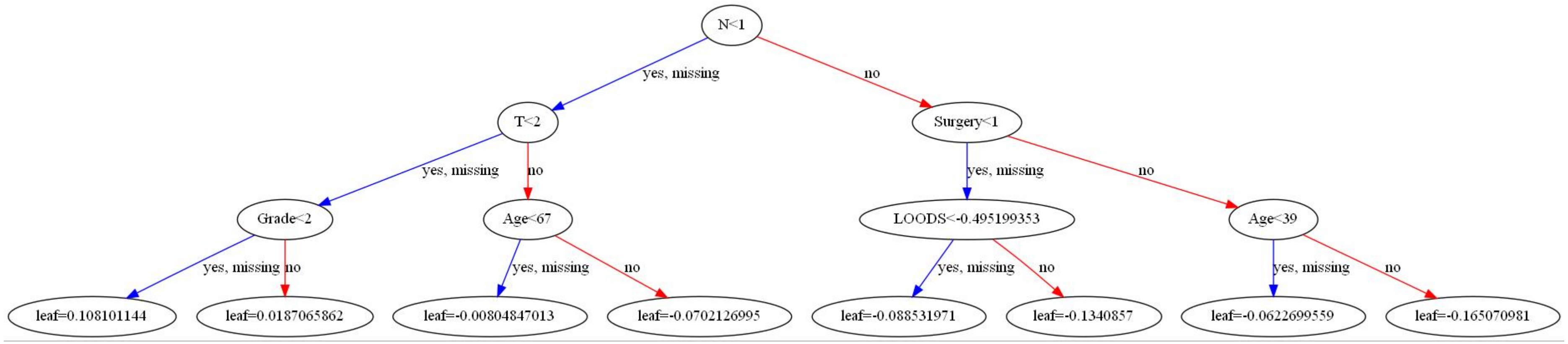

(H)

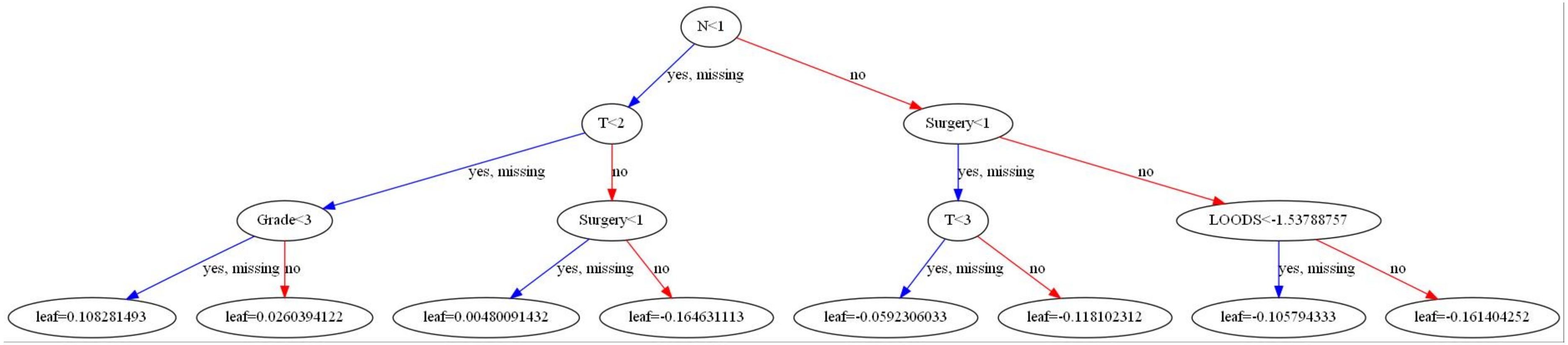

(I)

Supplementary Table S3

Parameters of the 7 machine learning methods

**Abbreviation**	**Method**	**Parameter**	**Programming language (version)**	**Function library (version)**
LR	LogisticRegression	penalty='l2',tol=1e4,C=1.0	Python(3.6.5)	scikit-learn (0.19.1)
SVM	SVC	C=1.0, kernel='rbf'	Python(3.6.5)	scikit-learn (0.19.1)
RF	RandomForestClassifier	n_estimators=10, criterion='gini', min_samples_split=2, min_samples_leaf=1	Python(3.6.5)	scikit-learn (0.19.1)
NBayes	BernoulliNB	alpha =1.0	Python(3.6.5)	scikit-learn (0.19.1)
DTree	DecisionTreeClassifier	criterion='gini',min_samples_split=2, min_samples_leaf=1 min_samples_split=2, min_samples_leaf=1,	Python(3.6.5)	scikit-learn (0.19.1)
XGB	XGBClassifier	max_depth=3, learning_rate=0.1,	Python(3.6.5)	xgboost (0.90)
KNN	KNeighborsClassifier	n_neighbors=5	Python(3.6.5)	scikit-learn (0.19.1)
